# Research on a Fast-Response Thermal Conductivity Sensor Based on Carbon Nanotube Modification

**DOI:** 10.3390/s18072191

**Published:** 2018-07-07

**Authors:** Hongquan Zhang, Bin Shen, Wenbin Hu, Xinlei Liu

**Affiliations:** 1 School of Automation, Harbin Engineering University, Harbin 150001, China; zhanghq1@126.com (H.Z.); huwenbin0119@hrbeu.edu.cn (W.H.); 2 School of Materials Science and Chemical Engineering, Harbin Engineering University, Harbin 150001, China; 3 School of Safety Engineering, Heilongjiang University of Science & Technology, Harbin 150022, China; lxl2020@163.com

**Keywords:** Al_2_O_3_, carbon nanotubes, thermal conductivity, gas sensor

## Abstract

Aiming at solving the slow-response problem of traditional bead-type thermal conductivity gas sensors, a fast-response thermal conductivity gas sensor can be made by using multiwalled carbon nanotubes (MWNTs), combined with the technology of carrier modification, to modify the performance of the sensor carrier. The carrier material, granular nanoscale γ-Al_2_O_3_/ZrO_2_, was synthesized by chemical precipitation, and its particle size was found to be 50–70 nm through SEM. After the carrier material was wet-incorporated into carbon nanotubes, the composite carrier γ-Al_2_O_3_/ZrO_2_/MWNTs was obtained. The results show that the designed thermal conductivity sensor has a fast response to methane gas, with a 90% response time of 7 s and a recovery time of 16 s. There is a good linear relationship between the sensor output and CH_4_ gas concentration, with an average sensitivity of 1.15 mV/1% CH_4_. Thus, the response speed of a thermal conductivity sensor can be enhanced by doping carbon nanotubes into γ-Al_2_O_3_/ZrO_2_.

## 1. Introduction

It is of great significance for the management of production safety to accurately detect the concentration of characteristic gases in complex gas mixtures [1]. In the coal industry, it is often necessary to detect the safety status of hazardous chemicals in real time, especially for highly flammable, explosive, poisonous, and harmful gases [2], such as methane at concentrations of 80–90%. At present, coal mine methane-detection technology for low concentrations under 5% is relatively mature, but for detecting methane concentrations greater than 5%, there is a lack of real-time online detection technology. Thus, this technology cannot timely reflect situations of gas emissions and outbursts during coal mining [3,4].

A thermal conductivity sensor is a kind of chemical sensor that has been used for gas detection and has the advantages of a wide detection range, stable performance, and long service lifetime without catalyst aging. Therefore, it has been widely used in detecting gaseous hazardous chemicals due to its high working reliability [5,6,7,8]. At present, it has been applied to mine gas-extraction systems with a low response speed. Modern thermal conductivity sensors have a bead-type structure, which has greater stability than the traditional structure of a hot-wire bare coil, but it has issues owing to its slow response [9,10]. The Ministry of Industry and Information Technology of PRC wishes to shorten the response time in order to quickly capture information regarding dangerous gases in a manner that delivers a stable performance, so as to provide the ideal detection method for safe production practices. Accelerating the response time is one of the main areas of interest in research on thermal conductivity gas sensors. The Al_2_O_3_ carrier covering the hot wire isolates the direct contact between the hot wire and air and increases the time of the heat exchange equilibrium between them. Improving the thermal conductivity and channel structure of the carrier will improve the sensor response time.

In this study, combined with the pore-forming technology of multiwalled carbon nanotubes, a thermal conductivity sensor based on the bead-type structure of hot-wire coated ceramic powder was designed and fabricated. This approach improves the permeability of the sensitive ceramic bead material and opens more gas transport channels. Therefore, the efficiency of gas heat exchange is increased, and the sensor response time is shortened [11,12]. The thermal conductivity sensor modified with carbon nanotubes was also tested and verified. The results indicate that the response time has been greatly shortened while still ensuring the overall performance of the sensor. This research is beneficial to the development of real-time detection technology for high-concentration methane in coal mines.

## 2. Experimental Materials and Methods

### 2.1. Elemental Structure of the Thermal Conductivity Gas Sensor

The thermal conductivity gas sensor is based on the principle that the total thermal conductivity of a mixture of gases varies with the content of the measured gas. It consists of two arms—the detecting element and the compensating element—that make up an electric bridge. When the thermal conductivity is larger than that of air, the resistance of the detector is reduced. Likewise, when the thermal conductivity is smaller than that of air, the resistance of the detection element becomes larger. In both cases, the output voltage of the bridge changes, and the change in voltage is directly proportional to the gas concentration. The compensation element provides temperature compensation.

**Figure 1:**
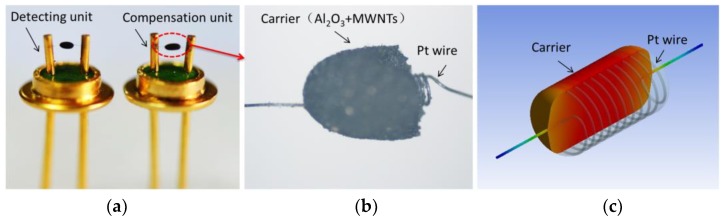
The element structure. (**a**) A pair of elements for the sensor. (**b**) Carrier bead structure. (**c**) Carrier bead cross-section.

Sensitive (detecting) and compensation units are a pair of elements with similar resistances (Figure 1a) that are made up of a Pt heating wire and a carrier (Figure 1b). The diameter of the Pt heating wire is 0.02 mm, and the number of winding loops is 10 (Figure 1c). Its static resistance is controlled at 3.0–3.5 Ω. The main components of the carrier are Al_2_O_3_ and multiwalled carbon nanotubes (MWNTs). The microstructure of the carrier is discussed and shown in Section 3.1, below.

### 2.2. Manufacturing Process of the Sensor

**Figure 2:**
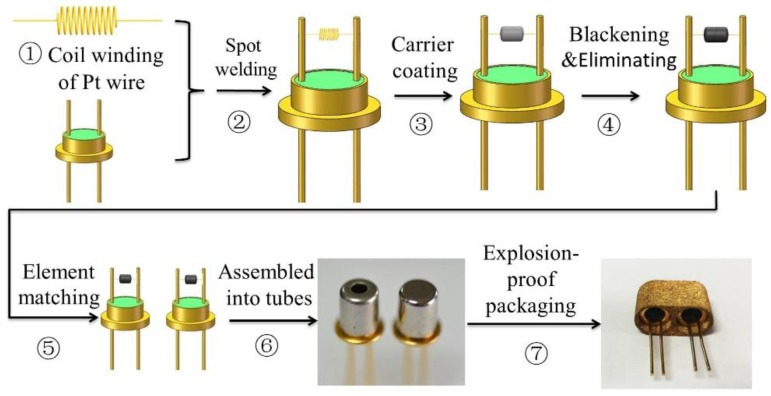
The manufacturing process of the sensor has seven main steps.

The thermal conductivity sensor contains a sensitive element and a compensation element, both of which have the same manufacturing method with different packaging and play a role in differential compensation. The sensor’s main manufacturing process includes seven steps, as shown in Figure 2.

Following the outline in Figure 2, step 1 is the coil winding of the Pt wire by using a wire-coiling machine. Step 2 is welding the Pt wire to the bottom seat by a spot welding machine. Step 3 is coating the mixed carrier of Al_2_O_3_ and MWNTs on the Pt wire. The elements should then be sintered. Step 4 is element blackening and the elimination of Pt catalytic performance. Step 5 is element matching based on the resistance values; elements with the same or similar resistance values will be paired as the two units of the sensor. Step 6 is standard tube packaging. The two elements are separately assembled into two standard tubes. One of them is a non-hermetic package and the other is hermetically packaged to compensate for the interference of ambient temperature on the sensitive component. Step 7 is the explosion-proof packaging of the sensor. The thermal conductivity sensor is made by assembling sensitive and compensation elements into the powder metallurgy cover to create an explosion-proof safety design.

The composition and microstructure of the sensor carrier are the key factors that restrict the performance of the sensor, including the response time. Compared to the manufacturing process of the traditional thermal gas sensor, there are three outstanding features of our manufacturing process: (1) the particle size of Al_2_O_3_ carrier reached the nanolevel; (2) the Al_2_O_3_ carrier was modified by MWNTs; and (3) the elements were blackened, and, further, we eliminated the Pt catalytic performance on methane. The detailed design is explained in the following, Section 2.3.

### 2.3. Key Process Design

#### 2.3.1. Carrier Material Design

γ-Al_2_O_3_ is a kind of catalytic carrier material with good activity, possessing an excellent load capacity for trace dopants and unique physical properties, such as a stable crystal form and a large surface area. It has a significant impact on air convection and conduction during the production of thermal conductivity sensor carriers [13,14]. Nanoparticles of γ-Al_2_O_3_ have a high specific surface area and form a large number of micro-nanoscale pores after doping with MWNTs. Some through-holes are beneficial to produce sensitive cells for thermal conductivity sensors.

Multiwalled carbon nanotubes (MWNTs) are prepared by LPCVD (low pressure chemical vapor deposition) with propylene as a carbon source and foaming nickel oxide as a catalyst. The flow catalyzed by propylene and N_2_ is 300 mL/min and 100 mL/min, respectively. Further, the catalytic cleavage reaction should be prepared at 600 °C [15].

In this paper, the nanoscale γ-Al_2_O_3_ ceramic ultrafine powder carrier material was prepared by chemical precipitation [16] and modified by adding 4–5% *w*/*W* nano-ZrO_2_ powder (50 nm, 99.99% purity, Macklin, Shanghai, China). Further, the MWNTs were wet-incorporated into the γ-Al_2_O_3_/ZrO_2_ powder at the ratio of 1% *w*/*W* for MWNTs/Al_2_O_3_ and then formed the composite carrier, γ-Al_2_O_3_/ZrO_2_/MWNTs.

#### 2.3.2. Blackening of Sensitive and Compensation Components

Nano-γ-Al_2_O_3_ has a high specific surface area and surface activity, and the carrier made from it has strong adsorption to polar molecules (including water molecules in the air). Since the grayscale of ceramic fired by the carrier γ-Al_2_O_3_/ZrO_2_/MWNTs is rather low, the heat radiation efficiency of the carrier will be strengthened, which will interfere with the detection of the convection effect for the sensor.

In this work, the carrier was changed from gray to black by nano-Pd particles formed by the impregnation of a pure palladium chloride acid solution (H_2_PtCl_6_·6H_2_O, analytically pure, Sinopharm Chemical Reagent Co., Ltd., Shanghai, China) and electrothermal decomposition. When the sensor is working, it can effectively reduce the thermal radiation efficiency of the carrier. In order to effectively reduce the catalytic effect of nano-Pd particles, a lead nitrate solution was impregnated with the surface of the black Pd particles, which formed desensitized lead monoxide after high-temperature decomposition. This finally formed the sensitive and compensation components of the thermal conductivity sensor with a small catalytic effect.

#### 2.3.3. Resistance Sintering and Pt Catalytic Performance Elimination

The first step of resistance sintering was to sinter the carrier powder slurry at a high temperature so as to combine the crystal particles and create micro-nano holes. Therefore, a stable, high specific surface area was formed with a certain degree of mechanical strength. Under the protection of high-purity nitrogen, the sensitive coil of the coated carrier was loaded with a DC (direct current) voltage and passed through a sintering current of 160 mA at about 600 °C for 60 min.

The second step was to thermally decompose the palladium chloride solution immersed in the carrier under the protection of high-purity nitrogen through a sintering current of 150 mA at about 550 °C for 30 min, thus blackening the sensitive and compensation components.

The third step was to thermally decompose the blackened carrier impregnated with the lead nitrate solution under the protection of high-purity nitrogen through a sintering current of 150 mA at about 550 °C for 30 min. After thermal decomposition, the lead nitrate solution formed PbO, which eliminated the catalytic effect of the palladium wire and palladium in the carrier. Therefore, there is only the thermal conductivity effect when the sensitive component is working. This step is very important. Although the working temperature of the thermal conductivity sensor will be controlled at 300 °C, the catalytic performance of palladium on methane still cannot be ignored at this temperature [17].

### 2.4. Test System Building and Performance Testing Method

**Figure 3:**
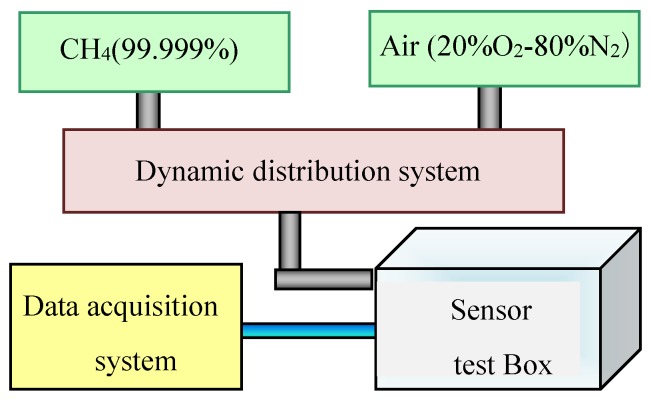
Dynamic gas testing system.

The test system consisted of a standard gas cylinder, an air source, a dynamic distribution system which can control the output CH_4_ concentration by MFC (mass flow controller), a sensor test box, a standard humidity generator installed in sensor test box, and a data acquisition system, as shown in Figure 3. The methane concentration in the standard gas cylinder was 99.999% and the air was dehumidified clean air.

**Figure 4:**
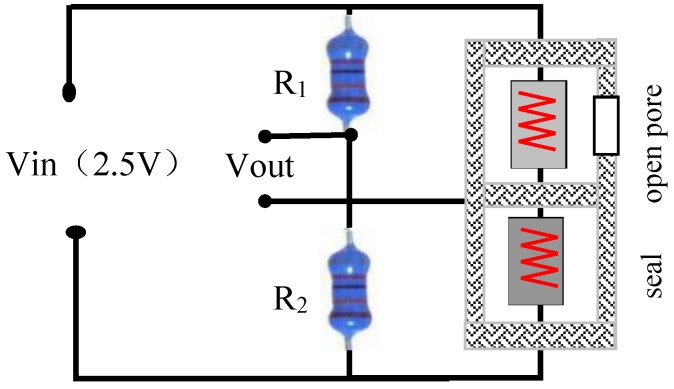
Detection circuit design, using a Wheatstone bridge.

The detection circuit was a typical Wheatstone bridge, as shown in Figure 4, that consisted of two 200 kΩ fixed resistors connected to a bridge arm on one side and the sensitive and compensation elements connected to a bridge arm on the other side. The two bridge arms were linked up to each other with constant voltage power. The detection circuit output different millivolt-level voltage values according to the variations of the gas components to be measured.

## 3. Results and Discussion

### 3.1. Microscopic Characterization of the Carbon Nanotube-Modified Al_2_O_3_ Carrier 

**Figure 5:**
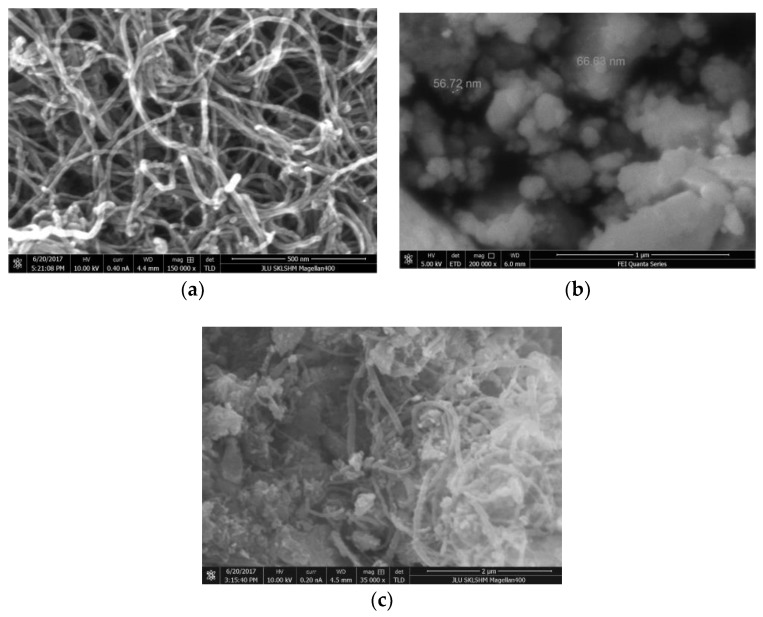
SEM analysis of the sensor carrier materials. (**a**) SEM of multiwalled carbon nanotubes (MWNTs). (**b**) SEM of the Al_2_O_3_ carrier particles. (**c**) SEM of the MWNTs mixed with the Al_2_O_3_ carrier.

The morphology of the multiwalled carbon nanotubes and the composite carrier material was observed and measured by an FEI Quanta FEG 250 field-emission scanning electron microscope with an operating voltage of 10 kV. Figure 5a shows that the outer diameter of the carbon nanotubes is 20–60 nm and their length is more than 2 µm, with a good draw ratio. Figure 5b shows that the size of the prepared Al_2_O_3_ carrier is about 60 nm. Figure 5c shows the carbon nanotubes and the Al_2_O_3_ carrier are entangled with each other, forming a channel within the carrier. The carbon nanotubes are mainly composed of sp2 hybrid mixed with sp3 hybrid nanotubular materials. Due to quantum physics effects, the network structures with different diameter MWNTs may create a special electrical property. In addition, carbon nanotubes are composed of carbon-carbon double bonds, hollow cages, and have closed topologies, so they have excellent mechanical and thermal properties [18].

**Figure 6:**
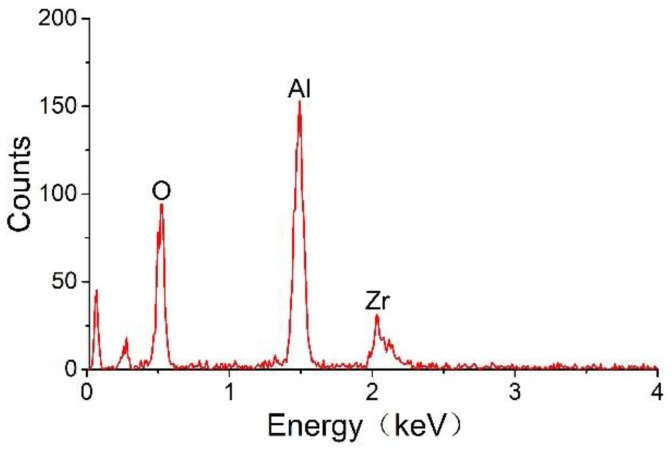
Energy spectrum of the sensor carrier.

Figure 6 indicates that the carrier mainly consists of Al_2_O_3_ and ZrO_2_ and that the expected ZrO_2_ component has been successfully mixed into the carrier. Here, ZrO_2_ is used as a carrier dopant to increase the thermal stability of the sensor.

### 3.2. Sensor Performance Test

#### 3.2.1. Output Characteristics of the Sensor

**Figure 7:**
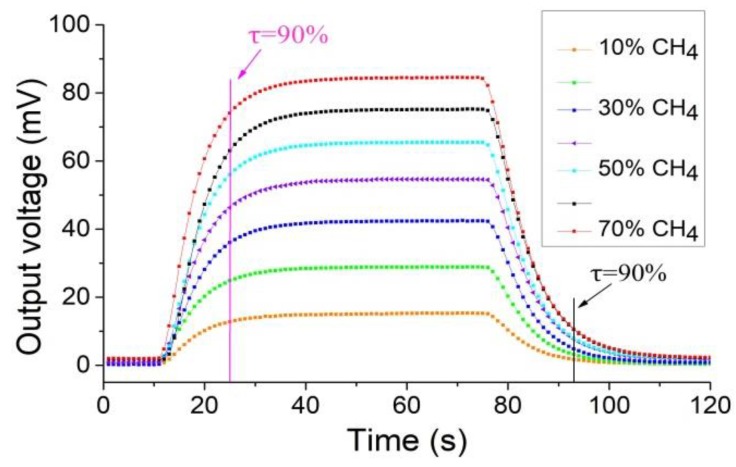
The response-recovery curve of the thermal conductivity sensor.

The response-recovery curve of the methane concentration was tested from 10% to 70% by controlling the methane concentration in the test system at an ambient temperature of 30 °C and humidity of 50% with the sensor’s operating voltage set to 2.5 V. The results are shown in Figure 7. The sensor has good response and recovery performance and the characteristics are similar at the different concentrations.

**Figure 8:**
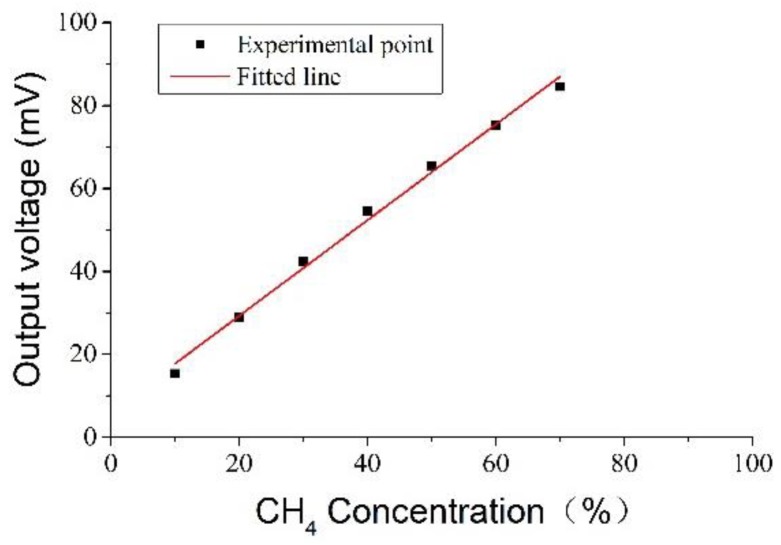
Sensor response to various methane concentrations with a linear fit.

A linear relationship between the output voltage of the sensor and the concentration of methane is shown in Figure 8. The best-fit formula is: y = 6.2094 + 1.1543x, *R*^2^ = 0.99275, where *R* is the linear correlation coefficient. The above formula indicates that the sensor output has a good linear relationship with methane concentration, and the average sensitivity is about 1.15 mV/1% CH_4_.

#### 3.2.2. The Effect of Ambient Temperature and Humidity on the Sensor’s Performance

**Figure 9:**
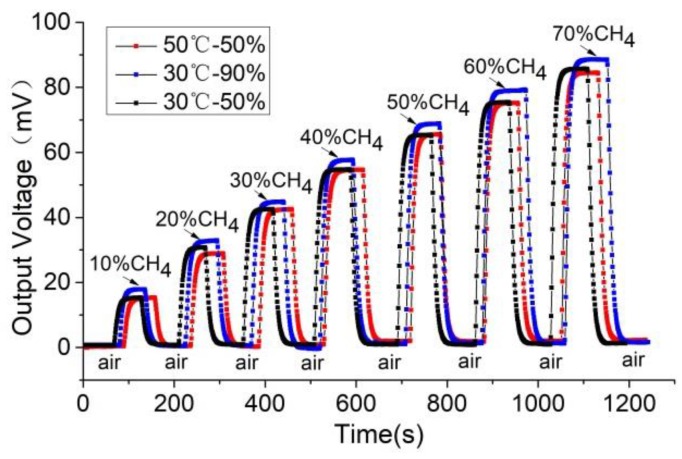
Response-recovery curves of the thermal conductivity sensor under different temperatures and humidity.

The methane concentration, ambient temperature, and humidity were controlled by the test system with a sensor operating voltage of 2.5 V under three conditions: 30 °C/humidity 50%, 30 °C/humidity 90%, and 50 °C/humidity 90%. The output voltage of the sensor was measured at methane concentrations of 10%, 20%, 30%, 40%, 50%, 60%, and 70%. From this, the response and recovery curve of the sensor to methane was obtained, as shown in Figure 9.

The above test results indicate that the change of the sensor’s ambient temperature from 30 °C to 50 °C has little impact on its response and recovery time and slightly reduces its response sensitivity (the average was less than 1 mV). The relative humidity change from 50% to 90% results in the response sensitivity increasing by an average of 3 mV. The effect of humidity on sensitivity was significantly greater than that of temperature.

#### 3.2.3. The Effect of Carbon Nanotube Doping on the Response Characteristics of the Sensor

The response time of the sensor includes both the time that the target gas takes to diffuse into the powder metallurgy package and the direct response time of the sensor to the gas. The specification of the powder metallurgy cover affects the gas diffusion time. Here, two specifications of 400 mesh and 200 mesh (which are used in commercial sensors), were chosen for gas testing. It is important to note that all the other performance tests of this sensor were under the 400-mesh powder metallurgy cover.

**Figure 10:**
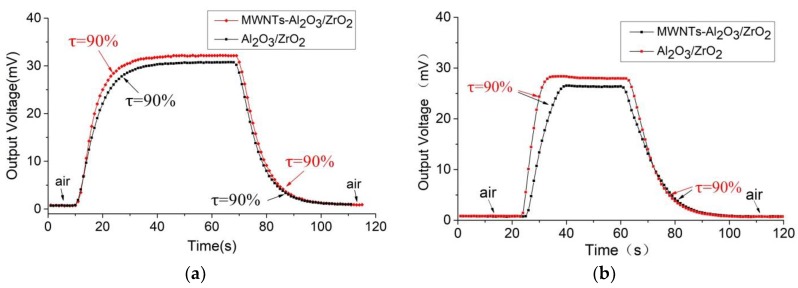
Response-recovery curves of different sensors for a methane concentration of 20%. Two powder metallurgy packaging materials are shown: (**a**) a 400-mesh package, and (**b**) a 200-mesh package.

Figure 10 shows the response and recovery curves of the thermal conductivity sensors using the carrier materials γ-Al_2_O_3_/ZrO_2_ and γ-Al_2_O_3_/ZrO_2_/MWNTs at a methane concentration of 20%. It can be seen that when the 400-mesh powder metallurgy cover is used, the sensitivity of the MWNT-doped thermal conductivity sensor is slightly higher than that of the undoped one. The 90% response time of the former sensor is 13 s and the recovery time is 16 s. The thermal conductivity sensor with the γ-Al_2_O_3_/ZrO_2_ carrier was tested under the same conditions and displays a response time of 17 s and a recovery time of 19 s. When the 200-mesh metallurgy powder cover is used, the sensitivity rule is the same. The response time of the thermal conductivity sensor with the carrier-doped MWNTs is 7 s and the recovery time is 16 s. The response time of the undoped thermal conductivity sensor is 10 s and the recovery time is 20 s.

Compared with commercial thermal conductivity gas sensors, such as the MD61 produced by the Winsen (Shanghai, China) company [19], with performance sensitivities of >10 mV/10% CH_4_, 90% response time ≤ 10 s, and 90% recovery time ≤ 30 s, the developed sensor has better performance. This suggests that the carbon nanotube doping has enhanced the response-recovery performance of the thermal conductivity sensor.

### 3.3. Fast-Response Mechanism Discussion of Thermal Conductivity Sensor

A thermal conductivity sensor is a kind of concentration sensor that responds to differences in the thermal conductivity coefficient between measured components of a gas mixture and a reference component, and it can thus sense the specific gas concentration in the environment. It can convert the information related to gas types and concentrations into the corresponding voltage values. The concentration of the measured gas can be obtained according to the relation between the output voltage of the sensor and the concentration of a component in mixed gas.

**Figure 11:**
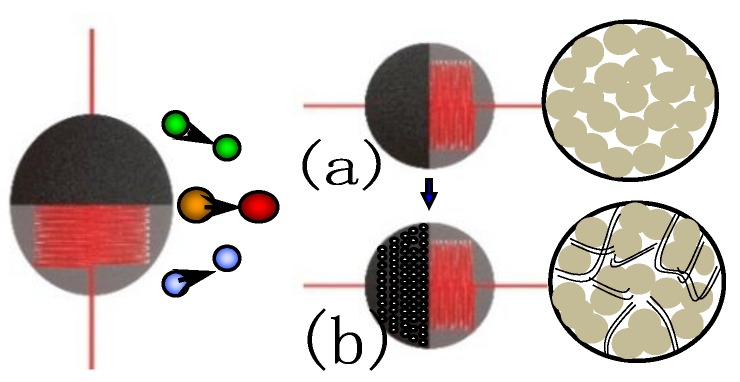
Schematic diagram of doped MWNT microchannel. (**a**) Carrier bead without MWNT. (**b**) Carrier bead doped MWNT.

Although granular γ-Al_2_O_3_/ZrO_2_ is in the nanoscale range and has a large surface area, there is still a large percentage of blind holes in the interior due to the carrier formation mechanism, as shown in Figure 11a. The imperviousness of the blind holes in the carrier can cause poor gas transport and lead to insufficient air exchange, which results in the slow response of the sensor and easily causes performance drift.

The catalytic carrier γ-Al_2_O_3_/ZrO_2_ modified by carbon nanotubes can effectively improve the electrical properties of the sensor. First, carbon nanotubes improve the microchannel of the carrier and make the measured gas transport more efficient [20], as shown in Figure 11b. Second, carbon nanotubes can improve the thermal conductivity of the carrier material, due to their high coefficient of thermal conductivity. Further, carbon nanotubes can also accelerate the heat exchange efficiency of measured gas on the sensitive carrier due to their larger aspect ratio. The resultant effect can greatly improve the response time of the sensor. In Figure 11, green, yellow, and blue balls are nitrogen, methane, and oxygen, respectively. Nitrogen and oxygen enter the carrier without changing the surface temperature of the carrier. However, when methane enters the carrier, it will affect the surface temperature of the carrier. The red ball indicates that the gas temperature has been changed.

## 4. Conclusions

(1) The carrier material γ-Al_2_O_3_/ZrO_2_ was modified by carbon nanotubes to make a thermal conductivity gas sensor. A test of methane under air conditions shows that the average sensitivity of this sensor is 11.5 mV/10% CH_4_ and that the sensor has good linearity. The effect of humidity on sensitivity is significantly greater than that of temperature.

(2) The sensor has good response and recovery performance for different methane concentrations, with the same response and recovery characteristics at different concentrations. The more compact the metallurgical powder cover, the longer the overall response time of the sensor, although this is not significant for the recovery time of the sensor. Under the 200-mesh specification, the 90% response time of the sensor can reach 7 s, and the 90% recovery time reaches 16 s, which is better than the traditional products and commercial sensors of the same type.

(3) The carrier γ-Al_2_O_3_/ZrO_2_ modified by carbon nanotubes improves its microstructure and creates more microporous channels, which makes the measured gas transport more efficiently. At the same time, carbon nanotubes have a higher thermal conductivity coefficient than the carrier γ-Al_2_O_3_/ZrO_2_. A composite carrier modified by carbon nanotubes can greatly improve the thermal conductivity, which is beneficial for the full heat exchange of the measured gas in the holes and facilitates the heat exchange efficiency of the measured gas on the carrier. Therefore, the sensor developed in this study shows the desired fast-response characteristics.
